# Hospital outcomes of acute COVID-19 infection among patients with neurological conditions: a single-center study

**DOI:** 10.3389/fneur.2024.1434046

**Published:** 2024-07-10

**Authors:** Adam Desouky, Venessa Fuentes, Chhitij Tiwari, Hikari Usui, Arthor H. Smith Ayala, Susan E. Wilson, Monica M. Diaz

**Affiliations:** ^1^Department of Epidemiology, Gillings School of Global Public Health, University of North Carolina at Chapel Hill, Chapel Hill, NC, United States; ^2^Department of Neurology, University of North Carolina at Chapel Hill School of Medicine, Chapel Hill, NC, United States

**Keywords:** COVID-19, SARS-CoV-2, neurological, in-hospital mortality, ventilation

## Abstract

**Background:**

Coronavirus disease 2019 (COVID-19) infection has been associated with severe neurological consequences, including stroke or seizures, and less severe neurological sequelae, including headaches, dizziness, and anosmia. Earlier COVID-19 variants were associated with high morbidity and mortality; however, knowledge of the impact of neurological conditions in the setting of COVID-19 on healthcare outcomes is limited. We sought to determine the impact of acute neurological conditions and acute COVID-19 infection on inpatient hospitalization outcomes.

**Methods:**

This was a retrospective, observational study of adult patients who were admitted to a large academic medical center in the Southeastern US between April 2020 and December 2021 with acute COVID-19 infection and a neurological diagnosis. Patient demographics, medical history, neurological diagnoses, and hospitalization outcomes were obtained from the medical record. Descriptive statistics and unadjusted and adjusted logistic regression analyses were performed.

**Results:**

Of the 1,387 patients included in this study, 27% died and 23% were kept under ventilation during hospitalization. The mean +/− standard deviation (SD) age was 64.6+/−16.9 years, with 52.8% women and 30.1% identifying as Black/African American. The most common neurological conditions included ischemic stroke (35.0%), movement disorder (12.0%), and hemorrhagic stroke (10.7%). In-hospital death was most common among those with epilepsy (*p* = 0.024), headache (*p* = 0.026), and dementia (*p* < 0.0001) compared to individuals without those conditions. Ventilation support was given more commonly to dementia patients (*p* = 0.020). Age was a significant risk factor for death (*p* < 0.001) and hospital length of stay (LOS) for ventilation (*p* < 0.001), but no neurological condition was a significant factor in adjusted logistic regression analyses.

**Discussion:**

Mortality was high in this study, with more than one-quarter of patients dying in the hospital. Death was the most common among those with epilepsy, headache, or dementia, but no neurological condition increased the risk of in-hospital mortality or ventilation. Future studies would determine the long-term neurological sequelae of those discharged from the hospital with COVID-19 and a neurological condition.

## Introduction

The central nervous system (CNS) and the peripheral nervous system (PNS) manifestations are evident in 36–82% of hospitalized adults with acute coronavirus disease 2019 (COVID-19) infection (1). Among patients with the severe acute respiratory syndrome coronavirus-2 (SARS-CoV-2) Omicron variant, the prevalence of the CNS and PNS symptoms was still as high as approximately 40% (2). Neurologic manifestations in the context of SARS-CoV-2 infection typically occur days after the onset of initial characteristic COVID-19 symptoms, including respiratory tract and systemic manifestations (3).

The emergence of neurologic signs and symptoms due to initial SARS-CoV-2 pre-Omicron variants may be attributable to various mechanisms, including direct damage by the virus, cytokine storm, hypercoagulable state, and molecular mimicry (4). It is thought that SARS-CoV-2 original variants may have demonstrated neurotropism (2, 5–7), leading to various neurological conditions observed during the pandemic onset. A recent study found elevated levels of neurofilament light (NfL) and glial fibrillary acidic protein (GFAP) among patients admitted for COVID-19, implying that dysregulations in both innate and adaptive immune responses are contributory to neurologic injury in the setting of COVID-19 (8). In the pre-Omicron era, prior to COVID-19 vaccination becoming widely available, acute encephalopathy, headaches, and stroke have been found to be common neurological syndromes among patients (3, 9).

Although the majority of individuals with acute COVID-19 infection who experience neurologic manifestations survive, the presence of neurologic signs or syndromes, such as acute encephalopathy or stroke, is associated with a higher in-hospital mortality risk (9, 10). Milder neurological symptoms, such as headache or diminished or loss of taste or smell, have been found to have a lower risk of in-hospital mortality (9). Furthermore, COVID-19-infected patients diagnosed after the emergence of the Omicron variant demonstrated lower disease severity and in-hospital mortality rates (11, 12).

The characterization of the effect of known neurological conditions on in-hospital outcomes is crucial to target treatments, particularly as COVID-19 evolves over time. In this study, we characterized the risk of neurological conditions on in-hospital mortality and ventilation among patients with acute COVID-19 infection during the pre-Omicron and Omicron waves of the COVID-19 pandemic.

## Materials and methods

### Study design and participants

We conducted a retrospective, observational study using electronic medical record review. Patients were identified using the Carolina Data Warehouse service, an inpatient registry of all patients admitted to our hospital, based on inclusion and exclusion criteria. We included all patients who were admitted to the University of North Carolina (UNC) Hospital at Chapel Hill, NC (USA) between 12 April 2020 and 28 December 2021. All patients were adults (18 years of age or older) who had acute COVID-19 infection and a known neurological condition based on ICD-10 diagnosis codes at the time of their hospitalization.

### Procedures

We extracted patient demographics, medical history, neurologic and non-neurological manifestations of COVID-19, and illness courses such as intensive care unit (ICU) admission and hospitalization outcomes, including in-hospital death and ventilation. Vaccination data were also available but were deemed unreliable, thus it was not included in these analyses. If a patient had two separate neurological diagnoses, each diagnosis was considered a separate outcome for the same patient (i.e., epilepsy or seizure and hemorrhagic stroke were counted as two separate outcomes for the same patient).

### Ethical approval

This study was approved by the UNC internal review board (IRB# 21–2036). Informed consent was not obtained given this was a retrospective chart review, and patients were not contacted.

### Statistical analysis

Descriptive statistics were performed on continuous variables [means and standard deviations (SDs)] and frequencies and percentages on categorical variables. Pearson’s chi-square tests of associations were calculated to compare the proportion of individuals with a neurological condition versus those without a neurological condition. Chi-square tests were adjusted for the number of comparisons performed. We used unadjusted logistic regression analyses to determine prevalence ratios and 95% confidence intervals (95% CI) for all outcome variables (in-hospital death and ventilation). Odds ratios were calculated under binomial distributions using SAS’s PROC GENMOD procedure for logit links. The errors of convergence were not present throughout the length of the study. We also performed multivariable logistic regression analyses that were adjusted for age, sex, race, and hospital length of stay. Statistical significance in this study was set at a *p*-value of <0.05 *a priori*. All statistical analyses conducted in this study were performed using SAS Studio 3.84 OnDemand for Academics.

## Results

In our study, we included 1,387 adults who met our inclusion criteria. Of these, 370 died during the hospital admission. The average length of stay in the hospital was a median of 7 days (interquartile range [IQR] 4, 13). The most common neurological conditions in the study were as follows: ischemic stroke (485 [35%]), hemorrhagic stroke (149 [10.7%]), and movement disorder (166 [12.0%]), among others (Table 1).

**Table 1:** Prevalence of death and ventilation by neurological condition (*N* = 1,387).

	Death (n [%])	No death (n [%])	Unadjusted and adjusted *p*-value for death outcome*	Ventilation (n [%])	No ventilation (n [%])	Unadjusted and adjusted *p*-value for Ventilation outcome*
Outcome total	370 (26.7)	1,017 (73.3)		319 (23.0)	1,068 (77.0)	
Ischemic Stroke	139 (28.7)	346 (71.3)	0.221/2.648	97 (20.0)	388 (80.0)	0.052/0.619
Hemorrhagic Stroke	44 (29.5)	105 (70.5)	0.404/4.853	25 (16.8)	124 (83.2)	0.056/0.673
Movement Disorder	42 (25.3)	124 (74.7)	0.669/8.033	41 (24.7)	125 (75.3)	0.579/6.950
Epilepsy or seizure	22 (18.0)	100 (82.0)	**0.024**/0.286	23 (18.9)	99 (81.2)	0.254/3.053
Headache	19 (17.6)	89 (82.41)	**0.026**/0.314	29 (26.9)	79 (73.2)	0.322/3.862
Dementia	40 (46.5)	46 (53.5)	**<0.0001/<0.05**	11 (12.8)	75 (87.2)	**0.020**/0.242

*Chi-square comparisons were performed between patients with each neurological condition and those without the neurological condition. *p*-values are shown unadjusted and adjusted for multiple comparisons. Bold values indicates *p*<0.05 significant.

Of all patients in the study, 319 (23%) required the use of bilevel positive airway pressure (BiPAP), or continuous positive airway pressure (CPAP), or both at one or more times during their hospital stay. Individuals with neurological conditions such as epilepsy/seizures, headaches, or dementia were more likely to die in the hospital compared to those with other neurological conditions. Patients with only dementia were more likely to be given ventilation support compared to those without dementia (Table 1).

In unadjusted logistic regression analyses, patients with dementia had a 2.6 times higher risk of dying in the hospital than those without dementia. Patients with either ischemic or hemorrhagic stroke had 1.17 times higher odds of in-hospital death than those without a stroke (*p* < 0.05). The odds of requiring ventilation were statistically significantly increased among those with movement disorders and hemorrhagic strokes (Table 2).

**Table 2:** Unadjusted odds ratios (ORs) of death or ventilation among patients with acute COVID-19 infection by neurological condition (*N* = 1,387).

	Total (*n*)	Death^*^ (*n*)	Odds ratios for death Outcome	95% Confidence intervals	Ventilation^**^ (*n*)	Odds ratios for ventilation Outcome	95% Confidence intervals
Neurological condition^†^							
Ischemic stroke	485	139	1.167	0.911–1.494	97	0.766	0.585–1.002
Hemorrhagic stroke	149	44	1.172	0.807–1.704	41	1.113	0.763–1.623
Movement disorder	166	42	0.922	0.636–1.338	29	1.252	0.802–1.954
Dementia	81	36	2.559	1.645–3.980	25	0.647	0.413–1.014
Epilepsy or seizure	122	22	0.580	0.360–0.935	23	0.761	0.474–1.219
Headache	108	19	0.564	0.339–0.940	10	0.473	0.248–0.902

^*^Death was defined regardless of the length of the hospital visits among those who had a neurological condition during inpatient hospital admission. ^**^Ventilation status was defined as having ever been treated with bilevel positive airway pressure (BiPAP), or continuous positive airway pressure (CPAP), or both during inpatient hospital admission. ^†^Some patients were diagnosed with more than one neurological diagnosis by a healthcare provider.

Adjusting for age, sex, race, and hospital length of stay, we found that no neurological condition increased the risk of in-hospital mortality or ventilation. Older age statistically significantly increased the risk of in-hospital death, while the longer hospital length of stay increased the risk of needing ventilation (Table 3).

**Table 3:** Multivariable analyses of effect the of neurological conditions among patients with acute COVID-19 infection on in-hospital death as an outcome (*N* = 1,387).

Neurological condition	OR^1^	95% CI^1^	*p*-value
Ischemic stroke			>0.9
Negative	—	—	
Positive	1.02	0.77, 1.33	
Movement disorder			0.7
Negative	—	—	
Positive	0.93	0.62, 1.36	
Hemorrhagic Stroke			0.7
Negative	—	—	
Positive	1.09	0.72, 1.63	
Epilepsy or seizure			0.4
Negative	—	—	
Positive	0.81	0.47, 1.33	
Headache			0.4
Negative	—	—	
Positive	0.80	0.46, 1.34	
Dementia			0.2
Negative	—	—	
Positive	1.40	0.87, 2.25	
Age at the time of encounter	1.04	1.03, 1.05	**<0.001**
Sex			0.2
Female	—	—	
Male	1.20	0.94, 1.54	
Race			0.12
American Indian or Alaska Native	—	—	
Asian	0.33	0.03, 3.22	
Black or African American	0.24	0.04, 1.13	
Other race	0.18	0.03, 0.90	
Prefer not to answer	0.00		
Unknown	0.26	0.02, 2.51	
White or Caucasian	0.18	0.03, 0.83	
Hospital length of stay	1.01	1.00, 1.02	0.053

^1^OR = Odds ratio, CI = confidence interval. Bold values indicates *p*<0.05 significant.

## Discussion

In our study among patients with a neurological condition diagnosed with acute COVID-19 during the pre-Omicron and Omicron waves of the pandemic, we found that no neurological condition increased the risk of in-hospital death after adjustment for age, sex, race, and hospital length of stay. However, in-hospital death was more common among those with epilepsy/seizures, headaches, or dementia compared to those without neurological conditions. Our results highlight that these diagnoses may be more associated with in-hospital death. Moreover, we found that epilepsy/seizure or headache diagnosis had a lower risk of in-hospital death. Older age and hospital length of stay may have more influence on in-hospital outcomes than neurological conditions (Table 4).

**Table 4:** Multivariable analyses of the effect of neurological conditions among patients with acute COVID-19 infection on ventilation as an outcome (*N* = 1,387).

Factors	OR^1^	95% CI^1^	*p*-value
Ischemic stroke			0.5
Negative	—	—	
Positive	0.91	0.67, 1.21	
Movement disorder			0.5
Negative	—	—	
Positive	1.14	0.76, 1.69	
Hemorrhagic stroke			0.077
Negative	—	—	
Positive	0.64	0.38, 1.05	
Epilepsy or seizure			0.4
Negative	—	—	
Positive	0.80	0.47, 1.32	
Headache			0.2
Negative	—	—	
Positive	1.33	0.82, 2.12	
Dementia			0.075
Negative	—	—	
Positive	0.55	0.26, 1.06	
Sex			0.6
Female	—	—	
Male	1.07	0.82, 1.40	
Race			0.5
American Indian or Alaska Native	—	—	
Asian	0.77	0.07, 7.94	
Black or African American	0.43	0.10, 2.15	
Other race	0.55	0.12, 2.89	
Prefer not to answer	292,912	0.00, NA	
Unknown	0.32	0.01, 3.51	
White or Caucasian	0.50	0.12, 2.50	
Age at encounter	0.99	0.99, 1.00	0.2
Total inpatient stay	1.04	1.03, 1.05	**<0.001**

^1^OR = Odds ratio, CI = confidence interval. Bold values indicates *p*<0.05 significant.

Sociodemographic factors are known to be associated with in-hospital outcomes in the setting of COVID-19 infection. Although initial studies showed that individuals of older age, male sex, and white race were at higher risk of neurologic manifestations and a worse prognosis (9, 13, 14), younger age and female sex have also been associated with an increased likelihood of neurological manifestations among patients infected with the Omicron variant (2). Another study demonstrated worse cognitive performance 6 months post-hospitalization due to COVID-19 among individuals identifying as Black (15). In our study, we found that older age increased the risk of in-hospital death, while longer hospital LOS increased the risk of ventilation, but no neurological condition increased the risk of these outcomes after adjustment for demographics and LOS.

Headache, dizziness, nausea, vomiting, confusion, anosmia, ageusia, and myalgia are among the most commonly reported neurologic symptoms experienced by COVID-19-infected individuals in both pre-Omicron and Omicron variants (2, 3, 10, 16, 17). Among younger individuals, a rise in neurologic symptoms, such as altered mental status and seizures, was also observed when the Omicron variant emerged (18, 19). This is consistent with the findings of another study, which stratified subjects into pre-Omicron and post-Omicron surge groups and demonstrated that encephalopathy is the most common neurologic diagnosis among both variants (11). The frequency of stroke and seizure was higher among post-Omicron surge patients compared to pre-Omicron patients (11). Our study was conducted during the pre-Omicron and Omicron waves and demonstrated that approximately 30% of patients with a stroke died in the hospital. Interestingly, we found that having a headache diagnosis lowered the risk of in-hospital death in multivariable analyses. One study found that those with pain syndromes also had a lower risk of in-hospital death compared to those without pain. One possible reason is that those who had worse COVID-19 severity (i.e., respiratory distress) may suffer from pain perception, and therefore, headaches may not be reported by those with worse COVID-19 severity (20).

Moreover, we found that having a diagnosis of epilepsy/seizure or headache carried a lower risk of death in multivariable analyses. This is different from prior studies that found an increased risk of death among people with epilepsy and COVID-19 infection compared to people without COVID-19 (a hazard ratio of 2.15 [95% CI 1.78–2.59]) (21). However, two other studies did not demonstrate an increased risk of in-hospital death among people with epilepsy and COVID-19 infection (22, 23). It is possible that in our studies, the small number of death events among the epilepsy group (*n* = 22) may not have been powered enough to detect an increased risk of death. Further studies would require chart review to determine reasons why particular patient characteristics among the group of patients with epilepsy may have been protective against death.

The course of disease among SARS-CoV-2-infected individuals appears to be largely influenced by pre-existing neurological disorders during the pre-Omicron era. For instance, individuals with pre-existing cerebrovascular disease tend to have worse outcomes such as lack of clinical improvement, development of acute respiratory distress syndrome (ARDS), need for ICU treatment, and symptom remission (14, 24, 25). Patients hospitalized with acute COVID-19 infection and known neurodegenerative diseases, such as dementia, parkinsonism, or multiple sclerosis, experienced altered mental status more often than those without neurodegenerative diseases (26). Although patients with neurodegenerative diseases typically demonstrate higher COVID-19 mortality rates due to older age (5), a comparison between matched groups of COVID-19 hospitalized patients with and without neurodegenerative diseases showed no significant difference in mortality, hospital length of stay, and ventilation when controlling for code status (26). In our study, we did not have access to code status, which may have influenced our results. Similar to prior findings, after controlling for age, those with dementia, as well as other neurological conditions, did not have an increased risk of in-hospital mortality or ventilation.

Our study has some limitations. First, we neither controlled for the length of time with a neurological condition nor did we have information on COVID-19 severity, neurological disease severity, or staging, given the diagnoses were based on ICD-10 codes. Patients who were more likely to have had a neurological condition for longer may be at higher risk of poor in-hospital outcomes. We were unable to distinguish the timing of the neurological condition, given the nature of the dataset; therefore, those who were on ventilation may have had an increase in the incidence of a neurological condition during their hospital stay. Third, those who were in the hospital for a longer LOS may be more likely to have poor outcomes, and those with a code status of do-not-resuscitate may not have been given ventilation support. We did not have access to coding status data, and vaccination status data were inconsistent; therefore, the ventilation and mortality outcome findings may have been influenced by data that we were unable to capture. We did not have vaccination data, but future research would account for the effects of vaccination status, mortality, and ventilation. Finally, this was a large retrospective study based on ICD-10 codes from a registry of all patients who were admitted to our hospital; therefore, some diagnoses might have been missed if coded differently.

## Conclusion

Among patients with acute COVID-19 infection and a neurological condition, both mortality and ventilation were high, each at approximately one-quarter. Death was the most common among those with epilepsy, headaches, or dementia, but no neurological condition increased the risk of in-hospital mortality or ventilation. Future studies would determine the long-term neurological sequelae of those discharged from the hospital with COVID-19 and a neurological condition. It would also determine how the severity or etiology of neurological illness (i.e., sub-types of dementia) could impact outcomes of COVID-19 infection and how thrombolysis among patients with ischemic stroke and acute COVID-19 infection may affect outcomes. Our study highlights that those with a neurological condition should be monitored closely for adverse outcomes during their hospital stay if diagnosed with a COVID-19 infection.

## Data availability statement

The raw data supporting the conclusions of this article will be made available by the authors, without undue reservation.

## Ethics statement

The studies involving humans were approved by University of North Carolina at Chapel Hill IRB. The studies were conducted in accordance with the local legislation and institutional requirements. The ethics committee/institutional review board waived the requirement of written informed consent for participation from the participants or the participants’ legal guardians/next of kin because This was a retrospective study using chart review. Our IRB accepted a HIPAA waiver and did not require informed consent for this study.

## Author contributions

AD: Data curation, Formal analysis, Writing – original draft, Writing – review & editing. VF: Data curation, Writing – original draft, Writing – review & editing. CT: Formal analysis, Writing – original draft, Writing – review & editing. HU: Data curation, Formal analysis, Writing – original draft, Writing – review & editing. AS: Data curation, Writing – original draft, Writing – review & editing. SW: Conceptualization, Visualization, Writing – original draft, Writing – review & editing. MD: Conceptualization, Investigation, Visualization, Writing – original draft, Writing – review & editing.
